# ‘The Doctor Made Clear His Utter Contempt of Me, and I Can Remember It Still’^1^: Unmarried Women’s Experiences of Accessing the Pill in Scotland *c.* 1968–1980

**DOI:** 10.1093/shm/hkae060

**Published:** 2025-03-19

**Authors:** Kristin Hay

**Affiliations:** Economic and Social History, University of Glasgow, Gilbert Scott Building (East Quadrangle), Room 627, Glasgow G12 8QQ, UK

**Keywords:** oral history, Scotland, contraception, religion, reproductive justice

## Abstract

In 1968, the barrier of marital status was removed from oral contraception. This meant that for the first time, unmarried women could legally access contraception for both social and medical reasons. As a biomedical drug, the pill required women to engage more frequently with the medical profession, purportedly redefining the patient–practitioner dynamic. However, despite the removal of legislative barriers to family planning services, societal attitudes towards the use of the pill by unmarried women continued to regulate individual behaviours and restrict their contraceptive choices. This was heightened in Scotland, which lagged behind the rest of mainland Britain in implementing family planning services. Using oral testimony, coupled with archival evidence, this article traces the implementation of family planning services by the Scottish state. It then examines unmarried women’s early experiences of accessing the pill, and the impact of societal attitudes, gender inequalities and medical hostilities on their reproductive autonomy.

In 2021, the Scottish Government unveiled their ‘Women’s Health Plan: A plan for 2021–2024.’^2^ Established in the 2019 Programme for Government, the purpose of the Women’s Health Plan was ‘underpinned by the acknowledgement that women face particular health inequalities and, in some cases, disadvantages because they are women.’^3^ The Scottish Women’s Health Plan was the first of its kind in mainland Britain, proceeded by comparable reports published for England and Wales in 2022. Among the six key strategic prioritises outlined by the plan was ‘improv[ing] access to abortion and contraception services.’^4^ This particular priority was identified in response to findings by the Royal College of Obstetricians and Gynaecologists in 2019, which showed that ‘across the UK 37 per cent of women cannot access contraception services locally’ and that ‘many women’s healthcare services are fragmented and difficult to access.’^5^

Consequently, the Scottish Women’s Health Plan underpinned the enduring issues of accessing contraceptive advice and appliances in Scotland, exactly sixty years after the introduction of the first oral contraceptive pill—Enovid—was into the National Health Service (NHS). The pill was first approved for use in Britain in 1961. It was stylish, discrete, non-intrusive, and had a ‘virtually 100 per cent’ effectiveness rate in preventing conception.^6^ By 1964, historian Hera Cook estimates that around 480,000 women across Britain were being regularly prescribed one of the fifteen brands of oral contraceptives available, each offering their own tailored balance of progestogen and oestrogen in order to prevent pregnancy.^7^

In the opening years of its inception, the pill was only available for married women for medical purposes, such as to prevent a medically dangerous pregnancy. Yet, gradually throughout the 1960s, permissive legislation was passed which removed the barriers to accessing contraception and made contraceptive advice and appliances the responsibility of individual local health authorities. By 1988, Britain was considered a pioneer in contraceptive availability, with American researchers commenting positively on the rapid uptake of birth control use in the country and attributing it to ‘the characteristics of British society and its health care system.’^8^ Their analysis indicated that both a social, as well as medical, liberalisation of attitudes was necessary in delivering successful contraceptive utilisation. Coinciding with changes in divorce laws, the partial legalisation of abortion, and a growing emergence of a purportedly distinctive, affluent youth culture, newspapers began to argue that ‘a relentless social and sexual revolution’ had taken place in Scotland and across Britain, wherein traditional attitudes towards sex, sexuality and family planning were forever eroded.

For Hera Cook, alongside Elizabeth Siegel Watkins and Andrea Tone, the pill, as the first biomedical, female-controlled contraceptive represented a turning point in attitudes towards birth control, and in particular women’s sexuality and reproductive autonomy.^9^ Moreover, in accessing the pill, women engaged with the medical profession like never before, becoming what Alex Mold has described as ‘patient-consumers’ within the health service.^10^ Although numerous forms of female-controlled contraceptives had existed prior to the 1960s, the pill’s detached and discrete style—owing to its literal separation from sexual and reproductive organs - embodied post-war modernity, and, it is argued, facilitated the elevation of family planning into a national public health priority.

However, whilst the beginning 1970s marked the end of legislative barriers to contraception, in practice women across Britain continued to struggle to access family planning services; particularly women who were unmarried. Despite having the legal right to use contraceptives, this cohort was often considered to be inherently youthful and in need of paternalistic guidance, moral surveillance, and regulation.^11^ These enduring barriers were heightened in Scotland, which, in 1969, was considered by Mass Observation, a prominent British social research organisation, as ‘other end of the moral scale’ in comparison with ‘London – the centre of permissive society.’^12^ Taboos around nonmarital sex had a significant impact on women’s reproductive autonomy and wider sexual and contraceptive choices. Whilst these taboos were not unique to Scotland, this article will show that a particularly Scottish articulation of conservative religious attitudes had a disproportionate impact on the development of family planning service provision in Scotland—directly limiting contraceptive availability. This sociocultural climate meant that, for many unmarried women, ‘going on the pill’ for the first time was a fraught and emotional interaction that required making tacit decisions around where to access contraception, followed by a careful negotiation of the power dynamics at play within a limited range of medical spaces.

Understanding how women accessed prescribed contraception is crucial to fully assess the impact of state-managed contraceptive services and the purportedly permissive shift towards sexual liberation in mid- to late-twentieth-century Britain. The significance of health inequalities within sexual and reproductive healthcare has been well-researched in America and, more recently, has become a growing area of historical interest in the UK. As Lesley Doyal suggests, this original delay could be the result of ‘the existence of a nationalised medical care system providing treatment free at source.’^13^ Whereas healthcare is privatised in the United States, the existence of the NHS and its valorisation within British culture has curtailed the critical interrogation into how far the health service has upheld its original aims: to provide comprehensive, universal and free healthcare to all. Since Doyal’s original assessment in 1994, a great deal of research has been conducted in Britain by historians such as Roger Davidson and Gayle Davis, Caroline Rusterholz, Anne Hanley, Jane O’Neill, Sally Sheldon and Clare Parker, who highlight the fraught relationship between family planning and the NHS and issues of accessibility, particularly in the context of abortion.^14^

More recently, research into the intersectional inequalities of access to and attitudes towards birth control have focussed on Scotland, which had, and continues to have, distinctive legal framework and healthcare infrastructures to England and Wales. Roger Davidson and Gayle Davis’ influential work charted the legal and political shifts that took place in post-war Scottish society relating to sex, sexuality and sexual health.^15^ Charlie Lynch, Jane O’Neill and Callum Brown, among others, have further emphasised Scotland’s distinctive cultural experience of the so-called Sexual Revolution.^16^ As a result, Scotland is a particularly nascent case study through which to examine changes to contraceptive access and its impact on individual lives.

Sexual and reproductive health research in Britain and globally have also been informed and impacted by the important work of SisterSong, a collective of Black feminist-activists from Atlanta, who in the 1990s developed and championed the framework of reproductive justice. Loretta Ross and Rickie Solinger—an activist and human rights theorist, respectively—define reproductive justice as ‘the right *not* to have a child; the right to have a child; and the right to parent children in safe and healthy environments.’ Central to obtaining reproductive justice is ‘access to specific, community-based resources, including high-quality health care,’ which, it is argued, is essential in obtaining ‘sexual autonomy and gender freedom for every human being.’^17^ In 2021, the Scottish Women’s Health Plan itself acknowledged that ‘for services to be truly accessible, it is vital that women have choice about how and when they access care,’ echoing the sentiments of feminist-informed healthcare.^18^ Yet, with the notable exception of Caroline Rusterholz and Jane O’Neill, research into access to contraception in the aftermath of the 1960s in Britain remains sparse in the current historiography.^19^ Consequently, building on the research outlined above, it becomes clear that an examination of the provision of contraception through the lens of reproductive justice is needed.

This article will combine extensive oral history research with archival research to explore women’s experiences of accessing the pill in 1970s Scotland. After Kate Fisher’s significant oral history of birth control practices in 2006, oral history methodologies have been applied in various contexts to elucidate the emotional and personal accounts of historic sociosexual change by historians, including recent works by Laura Kelly, David Geiringer and Lynn Abrams.^20^ Traditionally, oral history acts as a ‘recovery’ tool in historical research, providing accounts that would otherwise not be available in traditional archives.^21^ The oral histories in this article further provide a ‘bottom-up’ account of the introduction of family planning into the NHS and its impact on young women’s contraceptive practices, sexual behaviours and long-term sense of self. This research compares oral history accounts with archival research to examine the confluences and disjunctions between the documented past and individual experiences.

Thirty-one oral history interviews were conducted with women born between 1945 and 1965 who were living in Scotland during the second half of the twentieth century. Interviews were conducted both in-person and online between 2019 and 2021, including during the Covid-19 pandemic. Inspired by the practices of Kate Fisher and Simon Szreter, respondents were recruited through organised oral history workshops and guest lectures with over-50s organisations, including the University of the Third Age (U3A), local history societies, and the Men’s Shed movement.^22^ The women interviewed were all white British, heterosexual, and identified with the gender they were assigned at birth. Unless otherwise stated, all women were born and raised in Scotland. Respondents came from different regions in Scotland, providing insight into the impact of regional variability and different patterns of access and acceptability.

Respondents came from a variety of socioeconomic backgrounds: they were the children of coal miners, farmers, hairdressers and medical practitioners. However, most respondents attended further education as a result of unprecedented widening access to college and university in Scotland throughout the 1970s.^23^ As a result, these respondents were often afforded greater social mobility than their parents. Respondent occupations included a former bookkeeper, a dinner lady, a primary teacher, a chartered surveyor and an academic. This adds nuance to the analysis of class within this work: although over half of respondents reported a working-class upbringing, their access to further education and subsequent careers reflected a more middle-class lifestyle. This article will show that at different points in their lives, class impacted their reproductive and contraceptive choices in different ways.

The article will begin by exploring the implementation of family planning services in Scotland and the sociocultural and economic factors that influenced it. This will show that social stigma played a key role in the delayed provision of family planning services in Scotland created an invisible societal barrier to contraception. Attention will then turn to women’s engagement with medical spaces. It will explore their narratives of accessing medical contraception and the challenges they faced in negotiating with medical practitioners—both within the GP surgery and the family planning clinic.

The article blends oral history theory with the history of emotions, influenced by the work of Penny Summerfield, Sara Ahmed, David Nash and Anne-Marie Kilday, to examine how women felt about their interactions with medical professionals and the impact it had on their composure.^24^ A reproductive justice framework will also be applied throughout this article to highlight how women’s choice of birth control and healthcare provider were limited in Scotland in spite of the technological and legislative developments of the 1960s. This article ultimately thus calls into question the emancipatory effect of the ‘Sexual Revolution’. Crucially, it provides new and intimate insight into the lived realities of young, unmarried women seeking birth control throughout this period.

## The Provision of Family Planning in 1970s Scotland

The integration of family planning into the NHS in 1974 was considered a turning point in British attitudes towards the use of contraception. Between 1968 and 1974, the state gradually took control of the provision of contraceptive services and removed the restriction of marital status, enabling both married and unmarried women to access free family planning support on the NHS. In 1980, Audrey Leathard, a former chairperson of the FPA, described this process as a ‘rationalisation of family planning provision—a slow movement from confusion to order’: recognising family planning as essential healthcare and subsequently enshrining its place within the health service.^25^ However, following the legislative changes, women in Scotland continued to struggle to gain full contraceptive access throughout the 1970s and 1980s. Structural issues such as a lack of centralisation, a limited provision of family planning facilities, and regional disparities continued to play a role in birth control availability following NHS Reorganisation, influenced by social and cultural factors.

In order to understand the significance of the legislative changes, it is necessary to firstly examine the earlier infrastructure of family planning provision. Prior to the late 1960s, family planning services in Britain were delivered by specialised, charity-run clinics. The vast majority of these clinics were operated by the FPA, who at their height in 1969 provided 90 percent of family planning services in Scotland and was the second largest healthcare provider in the UK.^26^ However, the arrival of the oral contraceptive pill in 1961 accelerated pre-existing demands for state-supported family planning to combat a perceived ‘population explosion’: an explosion which was often disproportionately focussed on former British colonies, including India and Guyana.^27^ When the pill was rapidly adopted as a contraceptive of choice throughout the first half of the sixties, ‘the growing need’ for provision further prompted government action.^28^

The state’s role in family planning began in England and Wales with the NHS Family Planning Act of 1967, which compelled Local Authorities to make provisions for family planning advice and appliances either through the setting up of clinics or through the financial support of pre-existing services (such as through the FPA). The Bill was the first in the UK to formally sanction the state support of family planning. Scotland was excluded from the Bill in its early stages. However, following pressure from the government, the Health Services and Public Health Act of 1968 was passed, which accorded the same provisions for Local Authorities in Scotland.^29^ Significantly, the two Bills also allowed ‘contraception on social grounds’ with ‘no distinction…made between married and unmarried women.’^30^ Thus, the barrier of marital status was removed from contraceptive provision. Women could also be prescribed for the purposes of preventing conception without medical necessity. Despite this significant change, family planning provision would remain distinctive from the NHS until 1974, when the NHS Reorganisation Bill finally enshrined family planning into the health service. This time, with the exception of Northern Ireland, all countries in Britain were included in the Act.

Despite the changing legislation from the 1960s, accessing contraception in Scotland remained difficult. Respondents regularly recalled difficulties in accessing contraception during this period, including Shena, a retired teacher born in Parkhead, Glasgow, in 1953, who recalled: ‘it wasn’t available as freely as it should have been, there was definitely something going on.’^31^ Similarly, Jessie (b. 1949), a former primary teacher, reflected:

I think people have the view that the Sixties was this permissive society where we wore all flowered apparel and [were] sleeping around because the pill was available, but that wasn’t as readily available - although it was available it wasn’t as readily available as people thought it was.^32^

Jessie clearly contrasted the popular memory of the sixties with her experience of contraceptive inaccessibility, emphasising a disconnect between the two narratives. Her account emphasises that although the pill was available, prescription contraception was not wholly accessible. Indeed, as early as 1969, concerns were raised about the implementation of family planning provision by Scottish local authorities, with one MP calling it a ‘peculiar anomaly’ in the context of England and Wales.^33^ Shortly after the passing of the Health Services and Public Health Act 1968, a Scottish Office circular was released, recommending Local Authorities to delay implementing the clause specifically referring to family planning provision ‘in light of the present economic situation.’^34^ This circular led to a confusing legislative landscape, wherein individual Local Health Authorities were not *compelled* to implement family planning in line with the Act until 1970, leading to regional variations.^35^ In a 1969 House of Commons debate, some Members of Parliament suggested that the clause was being ‘artificially withheld through difficulties created by the [Scottish] state’.^36^ Edwin Brooks, architect of the Family Planning Act, called the circular a ‘dilitory [sic.] delaying tactic,’ stating that ‘the argument which lays stress on the economic situation is quite spurious.’ MP John Mackintosh of Berwick and East Lothian, who initiated the debate, similarly argued that ‘the economic case made by the Government falls to pieces’, leading him to ask: ‘what is wrong with Scotland…why is there a difference between Scotland and England on Family Planning?’^37^ Given that Scotland and England had, by this time, comparable laws relating to family planning and that doubt existed to the extent that economic factors were impeding implementation, Mackintosh’s question reflected an understanding that sociocultural factors played a role in Scotland’s delayed implementation.

Indeed, Scotland was considered one of the most hostile countries in mainland Britain towards state-supported family planning. This social climate has been viewed as distinctive within the context of England and Wales: as Roger Davidson and Gayle Davis suggest, ‘within the British context, the Scottish public was the least supportive of free birth control and a more proactive role by the state in the provision of family planning.’^38^ Whilst stigma towards contraceptive use was not unique to Scotland, there was a perception that religion played a more influential role in Scotland’s conservative moral values. The enduring centrality of religion in Scottish society and culture was reinforced by Callum Brown’s assessment that religious decline was slower in Scotland compared to the rest of mainland Britain.^39^

Mackintosh’s speech echoed these sentiments, referring to a ‘a strong religious group in Scotland with deep conscientious objections to family planning’, which he suggested led to a more fervent rejection to ‘sex on the rates’ in Scotland.^40^ Indeed, in a letter written in 1970 to William Ross, the Secretary of State for Scotland, one Mrs A. Lavery from Glasgow wrote:

I deplore deeply that fornication is encouraged among the unmarried by the giving of contraceptives and by the most evil of all the murder of the unborn…I do not see why we should follow in England’s wake…Scotland retains enough of its Christian principles to prevent it from sinking to the depths of permissiveness and evil.^41^

Mrs Lavery’s letter, written in response to Scotland’s changing family planning laws, epitomises the close association drawn between the availability of birth control and increasing promiscuity and moral degeneracy. Moreover, this was influenced by Mrs Lavery’s ‘Christian’ beliefs: an endurance of religious values which she perceived to add to the distinctiveness of Scotland and their neighbours in the south. Mrs Lavery’s letter was not an exceptional case, as religion played heavily into the Scottish debate around oral contraception for the unmarried. In 1967, Reverend William Still, from Aberdeen, criticised doctors for prescribing the pill to women ‘without even asking “if she has a wedding ring”’. In a letter to the *Aberdeen Evening Express,* Rev. Still wrote:

Christians now live in a post-Christian age in an increasingly Pagan society, and therefore in a missionary situation, we know what we must do – we must fight for our faith and its moral and spiritual foundations…God help us, who else will?^42^

Still’s dramatic and archaic language nevertheless highlights a correlation made between state provided family planning and a perceived decline in Scottish religiosity.

The backlash against the provision of oral contraception for unmarried women should be understood within the context of the rise of sexology during the post-war period and the influence of birth control pioneers, such as Marie Stopes, in redefining family planning as essential to maintaining a healthy, complimentary sex life within the confines of (implicitly Christian) marriage throughout the twentieth century.^43^ As David Geiringer shows, the religious sphere was not immune to changing cultural attitudes, as the Catholic Marriage Guidance Council’s ‘rapid expansion’ throughout the 1960s emphasised, in part, ‘an awareness of a new, ‘modern’ understanding of sexuality,’ and the need for mutual sexual satisfaction.^44^ Thus, the concept of sex exclusively for the purposes of procreation was in decline in both secular and non-secular spaces in post-war Britain. However, whilst family planning within marriage could be categorised as an acceptable shift in attitudes for the benefit of marital happiness at the start of the 1960s, the removal of marital status and the availability of contraception for the unmarried was considered a fundamental erosion of Christian values within the Scottish state. In 1968, the Catholic Church banned the use of artificial contraception with *Humanae Vitae.* Yet, whilst the protestant Church of England accepted contraceptive use ‘in the light of the same Christian principles’ from 1930, the Church of Scotland ‘condemn[ed] the use of contraceptives for such motives as indulgence, luxury, or a selfish desire to avoid the sacrifices which a family inevitably entails,’ until an explosive and controversial General Assembly in 1970.^45^ This permissive move by the Church was met with severe public hostility, and made headline news across Britain.^46^ Religion thus continued to impact societal attitudes towards the use of contraception in Scotland.

It is clear that religion played an influential role in Scottish society and culture, which in turn influenced the delayed implementation of family planning services. In 1969, Aberdeen’s Medical Officer of Health identified the ‘four enemies of family planning,’ as ‘Puritans, certain Catholics, traditionalists and penny-pinchers,’ emphasising an alleged role of religion in limiting access to services.^47^ In 1971, the chairman of Edinburgh City Council’s health committee rejected calls to make family planning services available to unmarried women, stating that ‘public money…should not be spent “in a manner many people consider to be offensive, degrading, corrupting and immoral.”’^48^ Also in 1971, Aberdeenshire Councillors opposed the setting up of a family planning clinic in the town of Keith, with Councillor George Watt stating to the local press that ‘I am opposed to this clinic on entirely moral grounds…I am certainly not convinced they don’t encourage promiscuity. I am positive they do.’^49^ These regional examples had a national impact. In 1979, a report into the organisation of Family Planning Services in Scotland found that ten out of the fifteen Health Boards in Scotland had failed to appoint a Coordinator for Family Planning, and four failed to provide any administrative support for the provision of services. Consequently, the report concluded that there was ‘no clear mechanism as to who directs the development of the services’ in Scotland.^50^ The 1979 report further remarked that in Scotland, ‘the overall picture presented is one of great variation and a lack of any substantial change in services from 1972’, further demonstrating the limited impact of legislation.^51^ These findings suggested that in spite of the transfer of family planning services in 1975, the relative autonomy of individual Health Boards and lack of centralisation facilitated delays to the implementation of robust family planning initiatives, in part due to social and cultural hostilities towards birth control.

These rejections demonstrate the societal factors, not economic factors, which influenced the implementation of the Health Services and Public Health Act. Although often aligned with ‘morality’, they were nevertheless imbued with conservative, implicitly Christian values. The conflation between the pill and ‘degradation’, positioned unmarried women accessing contraceptives as immoral and promiscuous. As a result, many respondents felt that going on the pill whilst unmarried was inherently wrong. This was demonstrated by Nora, a retired chef born and raised in Barra (a remote island in the Outer Hebrides to the West of Scotland) in 1947, who recalled:

I think it was generally thought it was for married people. I think it was more...It really wasn’t meant for single girls. Do you know what I mean? I think it was made for married couples. That’s what I thought.^52^

Nora was one of only a few respondents who waited until she was married to use contraception at age 26. Nora was 20 when the barrier of marital status was removed from contraceptive prescriptions, yet she continued to maintain this belief—emphasising that this attitude remained embedded in understandings of contraceptive use. This was further demonstrated in Irene (b. 1956)’s testimony. A social worker from Paisley, Renfrewshire, Irene recalled, ‘[being on the pill] was something almost shameful. A bit disgusting.’^53^ Her emotive language demonstrates that the use of the pill by unmarried women was not normalised in the 1970s. Being discovered to be using contraception could have a significant impact on respondents’ lives, as revealed in Beth’s (b. 1953) account, who commented that “it was a question of losing your reputation. That was the fear, was losing your reputation and being known as somebody who was loose.”^54^

These accounts reflected the individual need for ‘respectability’, which, as Sam Brewitt-Taylor notes, was central to ‘Britain’s Christian moral identity’ until the 1960s, when it was supplanted by a similar—yet more secular—narrative surrounding ‘personal morality.’^55^ The above accounts demonstrate this transitional, secularising phase in action. Despite the removal of legislative barriers to contraception, societal barriers continued to influence respondent’s contraceptive choices in Scotland. Consequently, accessing contraception became a tense experience for women in Scotland.

## Choosing a Contraceptive Provider

The confusing legislative landscape prior to 1974, coupled with respondent anxieties over the use of contraceptives whilst unmarried, meant that where women accessed contraception played a significant role in their sense of composure. As mentioned previously, clinic services provided the bulk of family planning prior to 1968, with GPs playing a lesser, supplemental role through private practice. However, following the NHS Reorganisation Act of 1974, all specialised family planning clinics were transferred to the NHS. From 1975, GPs began to provide family planning on the NHS, enticed by generous remuneration. Rather than diminishing the role of the clinic setting, the Department of Health and Social Security (DHSS) recognised the ‘special nature’ of family planning services and defended the right for people seeking contraception to choose the provider that they felt most comfortable with.^56^ As a result, between 1968 and 1980, women were increasingly able to “choose” to get contraception from either a clinic or their local GP. Some younger, unmarried women had an additional outlet: a university health service. Despite the DHSS acknowledgement that a range of providers was important and essential, in reality women’s “choices” depended individual understandings of availability and the local provision on offer.

Throughout the 1970s and beyond, the family planning clinic remained the contraceptive provider of choice for women in Britain. In a lecture given by the National Medical Officer to the Royal College of Physicians in 1982, it was revealed that although clinics represented only seven per cent of family planning outlets in Britain, they provided 40 per cent of contraceptive services, which they argued ‘must make a statement about the consumer acceptability of these clinics.’^57^ Moreover, in 1985, a consumer report by the Institute on Population Studies found that ‘clinics provide[d] a wider range of methods suiting a wider range of clients’ and that ‘women who choose clinics prefer the anonymity of the clinic setting…the specialise[d] knowledge and experience offered by the clinics…[and] the greater chance of seeing a woman doctor.’^58^ This was reflected in respondent testimonies. Maggie (b. 1953), who attended a clinic for all family planning services, recalled that it ‘wasn’t intimidating or off-putting’, and ‘all staffed by female doctors, which was nice.’^59^ The last factor—the gender of practitioner—was particularly as significant, as the medical profession continued to be highly male-dominated during this time. In 1974, around 75 per cent of all medical practitioners registered to the General Medical Register were men.^60^ The limited recollection suggests that visiting the clinic was an uneventful experience for Maggie, but the fact that she distinguished it from other providers indicates that she was aware that other services could not be so welcoming. This was echoed by other respondents, including Nora (b. 1947) and Jane (b. 1957), who both simply recalled that their clinic service ‘was good.’^61^

However, as the transfer of family planning under the umbrella of the NHS was completed, the number of family planning clinics in Scotland—and the rest of Britain—began to decline. Between 1975 and 1990, the number of patients seen in clinics in Scotland dropped dramatically from just over 126,500 to 109,500 over the course of fifteen years. In comparison, the number of patients going to GPs for family planning increased to just over 299,000 in the same period.^62^ Services were highly depended on geography, which often fell along urban and rural division lines. For example, most respondents who lived in Glasgow, Stirling or Edinburgh often had local access—and so utilised—family planning clinics. Those who lived in smaller towns—such as Barrhead and Dunblane—or in parts of the Highlands and Islands went to their local GP. Scotland also had disproportionately less clinics than in England, compounded by the fact that Scotland only had one Brook sexual health clinic. As demonstrated by Caroline Rusterholz, the Brook clinic provided a sexual health service dedicated to teenagers.^63^ Whilst this increase could be interpreted as a successful transition into an integrated family planning service, it contradicted the expressed guidance of the DHSS and limited women’s choice of service provider. Moreover, Nora and Maggie’s decision to go to a clinic appeared to be based on a lack of awareness that there was a choice to be made. As discussed previously, Nora believed that the pill could only be accessed from a clinic when married. Confusion around the law was also demonstrated in Maggie’s interview, as she commented:

I started using the pill, and it wasn’t that easy to get even for married women. you had to go to Claremont Terrace [FPA Clinic, Glasgow], or something. You couldn’t actually just get it from your doctor.^64^

Her account denotes a lack of awareness around both the GPs role in family planning and her ability to choose a provider. Maggie was raised in Ayr—around 38 miles from Glasgow city. Yet, she believed that clinics were the only option for her to access contraception and recalled travelling via train to access the pill, even after her marriage.

In areas where respondents did not have access to a clinic, women were reliant on GP provision. In stark contrast to the accounts of Maggie and those who only went to clinics, respondents who frequented their GP for family planning recalled the physical environment in unprompted and excessive detail, ultimately reflecting a negative attitude towards these spaces. This was demonstrated in Kate (b. 1963)’s testimony, who vividly recalled her GP’s surgery in Barrhead:

It was an old building and the waiting room was more like something like old fashioned kind of bus shelter. It was just horrible. It just had wooden bench seating round it, people would just sit there and queue….It was just this dreary building. It’s now knocked down. But it was just really, really old fashioned.^65^

The fact that Kate found it necessary to describe the GP surgery emphasises that she believed it was significant to her narrative. Finn Enke has written extensively on the importance of space, arguing that ‘sites…help explain how sexualities…are actually constructed, on the ground, in everyday experience.’^66^ Her negative language, coupled with her frequent references to the outdatedness of the building, suggests that the space was uncomfortable and at odds with what she was visiting the surgery for. As Elizabeth Siegel Watkins has shown, one reason that the pill was so appealing to women was its ‘aesthetic quality’: the pill was considered the panacea of modernity and technological advancement.^67^ The modern, clinical imagery of the oral contraceptive pill stood in direct opposition to the GP surgery. Indeed, another respondent, Ann (b. 1956), also discussed a sense of heightened visibility in her description of the GP surgery in Edinburgh:

I can still picture it still, it was…it was a big, big house. It’s like [what] you’d see in a film and it was a GP surgery. It was a big waiting room and so everybody knew who everybody was and you were called in, in front of everybody.^68^

Ann’s recollection highlights that the setting of the local GP clinic exacerbated her anxiety surrounding getting contraception from her family doctor. The fear associated with being called up to go in removed the safety of anonymity for many women and made them vulnerable to being recognised. This aligns with Martin Moore’s research into the development of the ‘waiting room’ during the 1970s. As he asserts, ‘concerns about patients’ emotional states…influenced postwar redesigns of GP premises,’ acknowledging the significance of the space in facilitating composure.^69^ Thus, her testimony provides an insight into why the built environment was so significant to respondents’ sense of composure. Visiting a doctor’s ‘home’ in order to obtain contraceptives represented a distinctive clash of ideologies and a site of conflict between the public and private.

For some respondents without immediate access to family planning clinics, contraception was often deemed inaccessible, in spite of GP provision. Agnes (b. 1963), a former housing officer from West Lothian, travelled 20 miles to Edinburgh in order to visit a family planning clinic. She described that her choice to go to the clinic rather than her GP ‘was all very...well planned and pre-meditated.’^70^ Agnes’ account highlights that having the choice of service provider was important to respondents and helped to maintain their composure and sense of autonomy when accessing contraceptives. Whilst she had, in theory, local access to contraceptives through her GP, her strong preference for a clinic service encouraged her to travel. Yet, travel was not always an option for women, particularly working-class women. Shena, for instance, reflected that ‘you tended to think locally. And [the nearest clinic] wasn’t easy to get to bus-wise,’ which left her reliant on the provision in her immediate area.^71^ The absence of a specific family planning clinic thus prevented some respondents from going on the pill altogether. Margaret (b. 1954) was born in Inverness and raised in the Outer Hebrides. As she states, the culture in her local community made her delay going on the pill until she went to university:

I couldn’t believe anyone in their right mind would go to a doctor in the Western Isles [Outer Hebrides] for contraceptives! I mean, yid be spotted going in! You know, [and they would say] “And why would you be going to the doctor? You’re only 17. Your mother’s no’ said a word to me about anything.”^72^

Her account further encapsulates the ways in which respectability—as regulated by the local community—continued to influence women’s contraceptive practices. Respondents believed that during this time being seen to be going to the doctors as a healthy young woman raised suspicion in their local community. This made going to the GP for the pill an impossible option for some respondents. Margaret’s attitude was echoed by Deborah (b. 1956), who recalled:

It was a funny time, it seemed to be fully available - except…the family doctor [knew] all about you and your family…and so no. So, I waited till I got over to the big city, yeah.^73^

Deborah’s account shows that she was aware that GPs could provide family planning, yet she delayed her use of contraceptives due to a lack of family planning clinics in her area. These testimonies show the importance of providers in women’s decision-making around contraception and the impact of varying clinic provisions across Scotland during this period.

As shown above, for some respondents going on the pill coincided with leaving home and entering further education. This was demonstrated by Deborah’s (b. 1956) account: ‘I was starting university in Edinburgh, and I was going to be nearly 19 and I thought, “Right, once I get there to the university [I’ll go on the pill]”’. Consequently, Deborah accessed contraceptives from the University Health Service which she romantically described during her interview:

It was called the Pill Centre with a capital ‘P’, that’s what all the girls called it… The university medical health centre was called the ‘Pill Centre’ because that’s what people were wanting it… it was exciting, and everything was new, and I was young and in love and on the pill!^74^

Her vibrant account suggests that distance from home, coupled with a sense of freedom afforded through student life, made accessing the pill more acceptable to Deborah. As shown, her account thus aligns more closely with the dominant narrative of the so-called ‘Swinging Sixties’. Class was an undeniable contributing factor: as Jane O’Neill shows, university doctors were more likely to provide contraceptives to women who were considered better educated and seemingly more capable of maintaining the regimen of oral contraceptives.^75^ Similarly, Agnes (b. 1963) also recalled:

I was away from home…[and] the doctor’s surgery was in the middle of campus. Where you just knew that the doctor would be dealing with everything like that. There was no embarrassment about it…you were seeing doctors that were used to dealing with students.^76^

Agnes’ account further shows that the proximity of the health service to the campus enabled some respondents to feel confident to access contraceptives with relative anonymity. University Health Services were thus viewed by respondents as more (sexually) liberal, and they benefitted from the distance from home, as well as the proximity to the dominant perceptions of middle-class student cultures.

Yet, Jane O’Neill has further highlighted that even in university settings, accessing the pill could remain a tenuous and fraught action for young, unmarried female students.^77^ Jessie (b. 1949) recalled one particular doctor during her time at university who, as she described, “[gave] lectures about sex before marriage and told them to keep it above the waist…it wasn’t as straightforward as a little ol’ pill.”^78^ This highlights that the intersection of gender continued to influence women’s experiences. Moreover, as has been shown, attending university, or indeed relocating for university, was not always possible for respondents. Particularly for working-class women living at home, they had no choice but to access prescription contraceptives from their doctor or seek alternative contraceptive options.

## The Patient/Practitioner Dynamic in the Consultation

As Jessie’s account above shows, anxiety around accessing the pill was caused not only by local provision, but also by the face-to-face interactions they would need to have with medical practitioners—particularly medical men. Since the pill was often prescribed for reasons other than a medical necessity, historians have argued that oral contraception subverted the traditional power-dynamic between patients and their doctors. Women seeking oral contraception had the potential to become ‘patient-consumers’. As Alex Mold states, the concept of the patient-consumer rose in earnest from the 1960s, concurrent to ‘the growing importance placed on individual patient autonomy; and, secondly, the development of consumerism within healthcare.’^79^ The oral contraceptive pill further encouraged patient consumerism within the NHS because, as Hera Cook notes, ‘the pill…stood apart from all other drugs because it was taken by healthy women for long periods of time.’^80^ As a result, otherwise fit women could assert themselves within the medical environment and access contraception for social reasons as well as medical purposes.

However, whilst on the surface women became more actively engaged in their own reproductive healthcare, so too did they become more aware of the complex negotiation required of them in medical spaces.

It emerged that the sense of ‘impossibility’ many respondents felt was the direct result of the attitudes of the GPs themselves towards family planning. This dichotomy was represented in a letter written to the *British Medical Journal* by a Dr J. Campbell Murdoch from Glasgow in 1972:

There are so many doctors who believe that obedience to the moral law of God…to hound such doctors out of contraceptive services because they do not do what the patient is supposed to want is a real suppression of information…a doctor must act within a framework of morality.^81^

This letter highlights again the influence of religious values on attitudes towards contraceptive provision for the unmarried. In the context of abortion, Gayle Davis has shown that medical practitioners were ‘reluctant’ to provide terminations for a number of both professional and moral reasons.^82^ This letter suggests that it was also the case in the context of contraception, as shown by the fact that the author felt it was the role of the doctor to ‘act…in their patient’s best interests when they advise her to contain her sexual experience.’^83^ This shows that, rather than opt-out of providing family planning, GPs would do so with the purpose of acting as a moral arbiter for their patients seeking contraceptives.

Respondents expressed awareness of the individualistic nature of contraceptive access and often recalled personal instances of hostility from their local GPs. This included Shena (b. 1953), who recalled the difficulty she and her friends had when getting birth control during their late teens. As she described:

The man [the doctor] was a bit of a horror. He’d no you have the prescription on the National Health. And this was the experience of a lot of my girlfriends as well…So, there was still that wee kind of restriction.^84^

Shena’s testimony is telling of the barriers that individual doctors could put in place to limit women’s access to oral contraception. As shown in her testimony, she directly refers her GP’s hostility as a limit to her contraceptive access. Shena was not the only respondent who was explicitly refused the pill, as Alison (b. 1964) was also refused by her GP in Elgin—a town in the north of Scotland—stating that:

This is quite a story, I actually went to my GP and he said no…I was pretty clueless, it hadn’t occurred to me to go anywhere other than my family GP, and he said no…I didn’t know what else to do really…the only thing I knew of was my GP.^85^

Alison’s account reflects the impact of the limited provision of family planning providers and the ways in which practitioners continued to assert professional power over women seeking family planning. Her sense of helplessness also shows the limitation of the dual provision of services: without a clinic, Alison reflected that she did not have any other option but to use condoms instead, restricting her choice and reproductive autonomy.^86^

Another concern raised by respondents was the threat of disclosure. Most respondents were aware that GPs were not supposed to break confidentiality, yet confusion existed around the extent to which it was *unethical* and the extent to which it was *illegal*. This is demonstrated in Margaret’s (b. 1954) testimony when she stated that ‘confidentiality and anonymity wasn’t the big thing it is now.’^87^ Whilst she knew that doctors had a responsibility to keep patient information private, during this period, her account shows that there was less awareness of the importance of data protection and the rights of the patient. This limited the impact of patient consumerism on Margaret’s life, as she lacked the information necessary to assert her health rights. In reality, discussions surrounding doctor’s ethical obligations in the context of birth control persisted throughout the 1970s and 1980s, with the repeated outcome being that it was malpractice for GPs to break confidentiality agreements under any circumstances—including with those under sixteen.^88^ Nevertheless, respondents were aware that disclosure was a common occurrence. Kate (b. 1963) recalled her sister’s doctor breaking confidentiality by telling her parents that she went for the pill:

He actually told my parents. I suppose at the time she could have said ‘he’s not allowed to do that.’ But instead, he told my parents, and she was just appalled about it…But he was just – he’d been the family doctor for years and he thought this was, you know, and he was Catholic…she got Hell from my parents.^89^

Significantly, Kate links the doctor’s choice to disclose to her parents to his Catholic faith, Her recollection further indicates that the consequences for being found out to be taking birth control were weighted more significantly than the unethical practice in the eyes of concerned parents. Consequently, a sense of powerlessness was evoked by many respondents. This was epitomised by Margaret (b. 1954), who reflected:

What wid I have done at the age of sixteen-and-a-half if the doctor had told my mum? I would’ve got a slap ‘round the ear and I probably would have been locked in the bedroom fir a quiet down. And my dad would’ve hit the roof. So that would have been the end of that. There wid be no complaint intended, but who wid I’ve complained to? And if I did, everybody would say, ‘Quite right. You should listen ti yer mother and father.’^90^

Margaret’s account clearly shows the sense of risk and vulnerability associated with going to the family doctor and the continued sense of paternalism and power attributed to GPs during this period. Her testimony further contrasts with one of the fundamental principles of patient consumerism as addressed by Alex Mold: that is, the right to complain. As Mold states, ‘the reimagining of the patient as a consumer…opened up a space where questioning medical authority…was possible.’ However, Mold also emphasises the limitations of this principle through an intersectional lens, as she states:

Complaining about medical care remained difficult and contentious…[and] only a minority of patients (predominantly those who were middle class and articulate) could [complain]…a situation that perpetuated existing inequalities.^91^

This emphasises that even those women who recognised the injustice of their treatment by GPs would have faced difficulties in asserting themselves through the complaints process. Moreover, whilst some respondents voiced opposition to the doctor’s power over them, other respondents accepted the status quo. This included Deborah (b. 1956), who commented:

The GP told you and you just did what you were told…[It was a] kind of working class, just that ‘obeying the GP’ thing, which is what we were brought up to do. We’d never have asked a question, or – [we were] always quite obedient, really!^92^

Deborah’s account emphasises the enduring power of practitioners and the limitations of patient consumerism, particularly when intersected with class as well as gender. Working-class women patients often lacked the confidence and/or awareness to challenge male GPs and assert their reproductive autonomy, believing in the authority of seemingly better-educated medical men.

Ultimately, the testimonies collected made clear that engaging with medical professionals was a tense experience which had a significant emotional effect on women’s composure. When asked how women managed to get the pill from hostile doctors, Shena (b. 1953) said: ‘it would be bravery. It would be about how brave you were.’^93^ Shena’s comment shows the emotional labour required of women challenging medical doctors and asserting their reproductive autonomy. Respondents often recalled going on the pill as resulting in a sense of guilt and shame which needed to be overcome in order to assert their reproductive autonomy. This emphasises the continued stigma surrounding birth control during this period and reinforces the social conventions which continued to limit women’s access to birth control. GP interactions had a direct impact on women’s sense of self. Carol’s (b. 1949) testimony presents an interesting example of this:

I remember going to see my doctor, my GP…I think it must have been the year before I got married, something like that, to ask for contraception and it was so difficult to get contraception at that time…He was really contemptuous. And it was only because I promised I was getting married to my boyfriend in the reasonably near future that he was prepared to prescribe. But the doctor made clear his utter contempt of me, and I can remember it still.^94^

Carol’s recollection highlights that negative interactions with doctors had a lasting impact on women’s memories and sense of self. Being made to feel chastised or looked down on had a significant effect on respondent testimonies. This was similarly represented in Louise’s (b. 1948) narrative:

I remember having to pluck up courage for every time I went to that health service because it was quite hostile, I felt…I mean, I was only 19. Nobody really talked to me like a human being.^95^

Louise’s narrative emphasises the fear and pressure felt by women seeking birth control during this period. Her comment that she required courage in order to go highlights the lack of agency experienced when engaging with medical professionals about contraception.

Sometimes, this sense of shame and threat of judgement was internalised and evoked by the respondents themselves. This emerged in Kirsty’s (b. 1958) testimony, who was particularly emotive during this point in her interview. Her account effectively encapsulates the impact of societal attitudes on access to contraception. Due to its significance, it has been written out in full:

I suppose it’s one of those most awful things, that while our parent’s generation were frowning on it so much and just saying, ‘Don’t do it, don’t do it,’ we were actually in such a situation that we didn’t have any access to it.

It’s so difficult to think now that I felt like in some way I might be judged because I wasn’t married and I was on the pill, so I almost felt like I didn’t want to go to my GP…there was this feeling that this was not right because I was on the pill so I was having sex and I wasn’t married, so what kind of woman was I?^96^

Kirsty’s affecting account highlights that whilst women had a number of reasons to be justifiably concerned about accessing contraception from GPs, social stigma further entrenched this guilt and concern over going on the pill. David Nash and Anne-Marie Kilday refer to this as ‘presumptive shame’, which they define as

The display of attitudes which indulge in a widespread believe that an act is, or more importantly remains, shameful, despite important elements of liberalisation inspired by both legislation and public opinion.^97^

Their definition accurately reflects Kirsty’s experience and the intense conflict that could be experienced by unmarried women when accessing oral contraception in the context of 1970s Scotland. Despite being legal, the act of going on the pill could thus lead to a deep-rooted and lasting emotional toll.

Whilst respondents frequently referenced the difficulties they encountered when accessing contraceptives—and the impact this had on their sense of self—they also found ways of navigating these barriers. This would often include informing other women of sympathetic doctors and informing them of family planning clinics. Edith, a retired teacher from Edinburgh (b. 1957), described the following telling example:

It was very dodgy…somebody I knew told me that there was a doctor in the West End [of Glasgow]…who was well known for giving out prescriptions to the pill to unmarried women…Fortunately for myself and how many other young women, we were able to get on the pill.^98^

Edith’s account highlights the ways in which women would share positive experiences with practitioners in order to enable others to access contraception. Maggie (b. 1953) also discussed this in her interview, as she recalled informing other women of the FPA clinic in Claremont Terrace, Glasgow:

We [were] all - no, recommending is too strong a word - but *helping* other women be aware of it being there. You know, some of the women I worked with, so that they might make use of its services.^99^

Despite the artificial barriers put in place, either societally or institutionally, women still found ways of sharing knowledge within their networks and encouraging contraceptive use.

Not only did women communicate preferable services, but they also shared ways to ensure they would be prescribed contraception within the medical consultation. For example, Sheena recalled her friends telling GPs that ‘they were having trouble with their periods’ in order to ‘get round’ the doctor’s questions and get on the pill.^100^ A more inventive tactic came from Liz’s (b. 1952) testimony, a trade unionist from Barrhead, who recalled knowing beforehand that her doctor preferred to prescribe to married women. As such, she explained that:

Everybody went, they put a ring on their finger and they said, ‘oh, I’m at university, I’m a student but we don’t want to have children just yet.’ What nonsense. And you got the pill.^101^

Similarly, Jessie’s (b. 1949) testimony revealed:

Girls would go to the clinic but they would go to Woolworths and buy a cheap gold band – well, it wouldn’t be gold, it’d be in brass or something – to wear on the wedding finger so that they could pretend to be married.^102^

Faking marriage was not a tactic unique to Scotland. As Elizabeth Siegel Watkins study of America states: ‘a dime-store wedding ring usually sufficed, but it might have required more nerve than most teenagers could muster.’^103^ Yet, the above examples, occurring across Scotland, highlight how widespread the phenomenon of performance was in accessing contraception in 1970s Scotland. These ‘performances’ further reflect similar actions taken by women in Gayle Davis and Jane O’Neill’s respective studies of accessing abortion in Scotland.^104^ In particular, Davis found that ‘the success of the patient in obtaining an abortion in such case depended on her ability to engage her particular doctor’s sympathy.’^105^ As respondent testimonies show, performance and sympathy was also required when accessing contraception and was pre-mediated by respondents.

The employment of performance in order to access contraception highlights that although they had the right to access the pill by law, women still had to navigate sociocultural barriers in order to assert their reproductive autonomy. Yet, David Nash and Anne-Marie Kilday refer to this as ‘anti-shame’, which they describe as ‘the concerted effort of individuals, caught in shameful situations…seeking to somehow fight back.’ The narratives explored here emphasise the use of ‘counter-shaming,’ where respondents knew they were being placed in an unfair and disadvantaged position by doctors who were acting inappropriately. In turn, they justified their “ruse” as an act of reclaiming power and agency.^106^ Nevertheless, the need to perform undoubtedly highlights the ways in which women continued to perpetuate cultural mores to enhance their chances of obtaining contraceptives in 1970s Scotland.

## Reproductive Justice and Scottish Women’s Experiences of Accessing Contraception in 1970s Scotland

Loretta Ross and Rickie Solinger argue that fallacy of ‘choice…disguises the ways that law, policies and public officials differently punish or reward the childbearing of different groups of women.’^107^ Despite the centrality of ‘the right to choose’ within global birth control movements, reproductive justice provides a holistic lens through which choice and body autonomy can be asserted not merely through legislation but by implementation, accessibility, and acceptability.

This article has shown that Scotland continues to act as an important and salient case study through which to analyse the implementation of permissive legislation surrounding contraception in the late 1960s, particularly through the lens of reproductive justice. The distinctive legislative framework and NHS in place in Scotland during this time allowed for a focussed yet generalisable analysis of the transition from the law to implementation in both rural and urban settings in mainland Britain. From the late 1960s, Scottish women had the legal right to choose a family planning provider. Yet, in reality, a postcode lottery existed, which limited their ability to assert their reproductive agency. Further research will likely demonstrate that this is not unique to Scotland in the mainland British context. Yet, this microcosmic example provides a framework for future comparative and complimentary works, both in Britain and further afield.

Furthermore, this article has shown that sociocultural hostilities towards the use of birth control for the unmarried—though not unique to Scotland—were heightened in the Scottish context and so played a more visible role in delays to the implementation of family planning services. Crucially, these attitudes were often influenced by the omnipresence of religious values in Scottish culture and social mores. This finding does not challenge secularisation narratives: the backlash to the pill emphasised the very real fears of prominent religious groups who recognised and reacted to the increasing secularisation of the Scottish state.^108^ Rather, the religious influence in debates around contraception in the late 1960s help to complicate the cultural process of secularisation. Respondents consistently reflected on the influence of religion on both societal attitudes towards contraceptive use and structural difficulties of access. Additionally, religious values in Scotland were explicitly considered stronger at the time, denoting a sense of Scottish exceptionalism during the permissive shifts in the late 1960s onwards.^109^

Respondent narratives of accessing contraception provide vital insight into attitudes towards contraception, its sociocultural meaning, and its long-term impact on respondent lives and women’s sense of self. The women who participated in this study, particularly those who did not have access to a family planning clinic, had very strong memories of engaging with medical professionals in medical spaces to access the pill, which stayed with them throughout their lives. Going ‘on the pill’ was not the same as going to the GP for an antibiotic. It was a site fraught with tension and negotiation, where women navigated several social and institutional barriers to contraceptive access before entering the waiting room—particularly when they were unmarried. These attitudes reflected gendered perceptions of morality and were influenced by religious values. Moreover, respondent’s sense of security was dependent on what they had local access to—issues of anonymity, confidentiality and disclosure were omnipresent in contraceptive access and could impact women’s sense of composure if they were compromised.

Although the power balance between patients and practitioners was seemingly redefined in the context of oral contraception and the 1970s more broadly, the endurance of medical paternalism, societal attitudes and gendered inequalities meant that women often had to perform in order to access contraceptives that they legally entitled to.^110^ They wore fake wedding bands, told doctors they were engaged, feigned menstruation problems or expressed relief when those problems were real and justified oral contraception. The importance of women sharing knowledge about sympathetic practitioners with one another was raised consistently throughout their testimonies and reflects the actions taken by women in places where birth control remained illegal or illicit.^111^ However, despite the myriad of ways that women tried to circumvent these institutional barriers, the threat—and lived reality—of refusal and disclosure continued to impact their reproductive autonomy. Even after finally obtaining their contraceptive of choice, women continued to feel a sense of shame and judgement, highlighting that contraception was not normalised for unmarried women in the 1970s.

By considering women’s contraceptive access in Scotland through the lens of reproductive justice, we see how women’s choice and reproductive autonomy were impacted by issues of access: both in terms of location but also in terms of sociocultural barriers which continued to restrict family planning for the unmarried. Oral history testimony also reveals that accessing contraception was a very emotive experience and was a significant moment in most women’s life histories. Contraception was an omnipresent feature in heterosexual women’s lives, which often required careful consideration and medical surveillance. It also required asserting themselves within a patriarchal medical setting and dealing with the fallout of shame, stigma and judgement that came with it.

These experiences highlight the enduring gender inequalities within healthcare and the often-overlooked emotional labour heterosexual women went through in order to assert their reproductive autonomy. The history of family planning in Scotland has acute relevance to the present day and adds urgency to contemporary commitments to providing comprehensive, trauma-informed sexual and reproductive healthcare in the present. It also emphasises the need to engage with patient experiences of these health services and their preferences for service provision.

## Data Availability

The archival data supporting the findings reported in this paper are openly available from the Glasgow Women’s Library, the Lothian Health Services Archive, the Mitchell Library, National Records Scotland, and the Wellcome Library. Due to ethical requirements, the oral history data supporting the findings reported in this paper are not currently openly available. Please contact the author for further information. For the purpose of open access, the author(s) has applied a Creative Commons Attribution (CC BY) licence to any Author Accepted Manuscript version arising from this submission.

## Footnotes

2
*Women’s Health Plan: A Plan for 2021–2024* (Scottish Government, 2021), 8

3
*Ibid.*, 25.

4
*Ibid.*, 8.

5
*Better for Women* (Royal College of Obstetricians and Gynaecologists, 2019), 38.

6 FPA pamphlet, ‘The Pill’ (1973) [Glasgow Women’s Library (hereafter GWL), FP-11-6] For more on the history of the oral contraceptive pill and its initial reception, see L. Marks, *Sexual Chemistry: A History of the Contraceptive Pill* (New Haven: Yale University Press, 2001); E.S. Watkins, *On the Pill, A Social History of Oral Contraceptives 1950–1970* (London: John Hopkins, 1998); A. Tone, *Devices and Desires: A History of Contraceptives in America* (New York: Hill and Wang, 2002).

7 H. Cook, *The Long Sexual Revolution: English Women, Sex, and Contraception: 1800–1975* (Oxford: Oxford University Press, 2004), 281.

8 J. Silverman and E. F. Jones, ‘The Delivery of Family Planning and Health Services in Great Britain’, *Family Planning Perspectives*, 1988, 20, 68–74.

9 See Tone, *Devices and Desires:* Cook, *The Long Sexual Revolution.*

10 A. Mold, *Making the Patient-Consumer: Patient Organisations and Health Consumerism in Britain* (Manchester: Manchester University Press, 2016).

11 For more on the relationship between marital status and changing perceptions of adulthood in the 1970s, see K. Hay, ‘‘The pill for the unmarried girl is hardly going to improve her character’: the impact of changing sexual behaviours on the construction of adulthood in Scotland, c. 1968–1980’ in L. Tisdall and M. Cannon, eds, *Adulthood in Britain and the United States from 1350 to Generation Z* (forthcoming, 2024).

12 MO Press Release, ‘Morals aren’t what they used to be’ (1969) [WL SA/FPA/C/E/16/2/3].

13 L. Doyal, ‘Women, Health and the Sexual Division of Labor: A Case Study of the Women’s Health Movement in Britain’, in E. Fee and N. Krieger, eds, *Women’s Health, Politics, and Power: Essays on Sex/Gender, Medicine, and Public Health* (Amityville: Beywood, 1994).

14 Davidson and Davis, *The Sexual State: Sexuality and Scottish Governance 1950–80* (Edinburgh: Edinburgh University Press, 2012); G. Davis, ‘The Great Divide: The Policy and Practice of Abortion in 1960s Scotland’, online publication, Royal College of Physicians of Edinburgh (2005); A. Hanley, ‘Migration, Racism and Sexual Health in Postwar Britain’, *History Workshop Journal*, 2022; J. O’Neill, ‘‘Abortion Games’: The Negotiation of Termination Decisions in Post-1967 Britain’, *Journal of the Historical Association*, 2018; J. O’Neill, ‘‘Education not fornication?’: Sexual Morality Among Students in Scotland, 1955–1975’ in J. Burkett, ed, *Students in Twentieth-Century Britain and Ireland* (Cham: Palgrave MacMillan, 2018); G. Davis, J. O’Neill, C. Parker and S. Sheldon, ‘All Aboard the “Abortion Express”’: A Historical Geography of the 1967 Abortion Act, in G. Davis and C. Sethna, eds, *Abortion across Borders: Transnational Travel and Access to Abortion Services* (Maryland: Johns Hopkins, 2019).

15 Davidson and Davis, *The Sexual State.*

16 O’Neill, ‘Education Not Fornication’; C. Lynch, ‘Moral Panic in the Industrial Town: Teenage ‘Deviancy’ and Religious Crisis in Central Scotland, c. 1968–9’, *Twentieth Century British History*, 2021, 32, 371–91; C. G. Brown, *Religion and the Demographic Revolution: Women and Secularisation in Canada, Ireland, UK and USA Since the 1960s* (Suffolk: Boydell, 2012).

17 Ross and Solinger, *Reproductive Justice: An Introduction* (California: University of California Press, 2017), 9.

18 ‘Women’s Health Plan’, 20.

19 C. Rusterholz, *Responsible Pleasure: The Brook Advisory Centres and Youth Sexuality in Postwar Britain* (Oxford: Oxford University Press, 2024); O’Neill, ‘Education Not Fornication’.

20 K. Fisher, *Birth Control, Sex and Marriage in Britain* (Oxford: Oxford University Press, 2006); L. Kelly, *Contraception in Modern Ireland: A Social History 1922-1992**(Cambridge: Cambridge University Press, 2023)*; L. Abrams, ‘Talking About Feminism: Reconciling Fragmented Narratives with the Feminist Research Frame’, in K. Sringley, S. Zembrzycki and F. Iacovetta, eds, *Beyond Women’s Words: Feminisms and the Practices of Oral History in the Twenty-First Century* (Abingdon: Routledge, 2018); D. Geiringer, *The Pope and the Pill: Sex, Catholicism and Women in Postwar England* (Manchester: Manchester University Press, 2019).

21 P. Thompson, ‘The Voice of the Past: Oral History’, in R. Perks and P. Thompson, eds, *The Oral History Reader*, 3rd edn (London: Taylor and Francis, 2015), 34.

22 Fisher, *Birth control* and S. Szreter and K. Fisher, *Sex Before the Sexual Revolution*: *Intimate Life in England 1918–1963* (Cambridge: Cambridge University Press, 2010).

23 For more on Scotland’s approach to higher education in the second half of the twentieth century, see L. Paterson, *Scottish Education in the Twentieth Century* (Edinburgh: Edinburgh University Press, 2003).

24 P. Summerfield, ‘Culture and Composure: Creating Narratives of the Gendered Self in Oral History Interviews,’ *Cultural and Social History*, 2004, 1; S. Ahmed, *The Cultural Politics of Emotion* (Edinburgh: Edinburgh University Press, 2014); D. Nash and A. M. Kilday, *Shame and Modernity in Britain: 1890 to the Present* (London: Palgrave Macmillan, 2017).

25 A. Leathard, *The Fight for Family Planning: The Development of Family Planning Services in Britain, 1921–1974* (London: Macmillan, 1980), 157.

26 ‘Annual General Meeting, North of Scotland Branch: Chairman’s Address’, (1969) [ML, HB77/3/12].

27 HL Deb 28 June 1961, vol 232, https://hansard.parliament.uk/Lords/1961-06-28/debates/b6ab29bc-5688-4a12-8392-68fa9e426bcf/RegulationOfBirthAndWorldPeace [accessed 21 Sept 2023].

28 HC WA 26 May 1966, vol 713, https://hansard.parliament.uk/Commons/1965-05-26/debates/1bff722e-4c30-4c70-b517-698b295a7bf2/OralContraceptives [accessed 21 Sept 2023].

29 Health Services and Public Health Act 1968, https://www.legislation.gov.uk/ukpga/1968/46/contents [accessed 16 Sept 2022]. For more information on the legislation, see Davison and Davis, *the Sexual State.*

30 HL 19 June 1967, vol 283, https://hansard.parliament.uk/Lords/1967-06-19/debates/d4859046-1c41-4353-bee2-9c326881cec9/NationalHealthService(FamilyPlanning)Bill [accessed 21 Sept 2023].

31 Interview with Shena (23 Jul 2019), 30.

32 Interview with Jessie (9 Nov 2020), 4.

33 HC Deb 23 July 1969, vol 787, https://hansard.parliament.uk/Commons/1969-07-23/debates/be4f299b-f37e-4f3f-9094-55d32347b48a/Scotland(FamilyPlanning) [accessed 22 Sept 2023].

34 HC Deb 23 July 1969, vol 787.

35 ‘Family Planning in Scotland’ [NRS HH61/1208].

36
*Ibid.*

37
*Ibid.*

38 Davidson and Davis, *The Sexual State*, 144.

39 Brown, *Religion and the Demographic Revolution*, 93.

40 HC Deb 23 July 1969, vol 787.

41 ‘Letter to Scottish Home and health Department from Miss. A. Lavery, Glasgow’, (21 Dec 1970) [NRS, HH61/1208].

42 ‘Christ Gave No Licence to Sin’ *Aberdeen Evening Express* (19 Dec 1967) Readership of the *Evening Express* was around 81,000, making it one of the largest regional newspapers in Scotland. See J G. Kellas, *The Scottish Political System* (Cambridge: Cambridge University Press: 2011), 198.

43 See Hall, *Sex, Gender and Social Change* for more information on the significance of Marie Stopes.

44 Geiringer, *The Pope and the Pill*, 113.

45 ‘The Lambeth Conference: Resolutions Archive from 1930’, *Anglican Communion Office*, 2005, ‘Religious Attitudes to Contraception: Church of Scotland’, 1967 [WL, SA/FPA/C/E/16/4/6].

46 ‘Kirk Protest Over Policy on Pill’, *The Times* (26 May 1970).

47 ‘Birth Control: Doctor Hits Out’, *Scottish Daily Express* (9 Dec 1969).

48 ‘Edinburgh and Family Planning’, *Aberdeen Press and Journal* (19 Feb 1971).

49 ‘Council Face Split Over Clinic Plan’, *Glasgow Herald* (17 Feb 1971) [NRS, HH61/1208].

50 ‘FP Services, Present Provision: Outcome of Questionnaire’ (1979) [NRS, HH98/135].

51 ‘FP Services, Present Provision’.

52 Interview with Nora (26 Jul 2019), 8.

53 Interview with Irene (19 Jul 2021), 12.

54 Interview with Beth (19 Nov 2019), 3.

55 Brewitt-Taylor, *Christian Radicalism*, 178.

56 Cited in Silverman and Jones, ‘The Delivery of Family Planning and Health Services in Great Britain’, 69.

57 ‘Extract from Lecture Given by National Medical Officer to the RCP Faculty of Community Medicine’, 1982 [WT, SA/FPA/C/F/8/9/7].

58 FPA, ‘Press Release: Consumer Report on Family Planning Services’, Nov 1985 [WL SA/FPS/C/E/16/4/3].

59 Interview with Maggie (10 Jul 2019), 11–13.

60 T. W. Lambert, ‘Doctors Who Qualified in the UK Between 1974 and 1993: Gender, Nationality, Marital Status and Family Information’, *Medical Education* 2002, 32, 533.

61 Nora p. 8, Interview with Jane (29 Oct 2020), 19.

62 FPA, ‘Fact File 1B: Use of FP Services in the UK’, Aug 1992 [LHSA GD22/6/2/1].

63 For more on Brook, see Rusterholz ‘Youth, Responsibility’.

64 Maggie, 3–11.

65 Interview with Kate (16 Jul 2019), 15.

66 See F. Enke, *Finding the Movement: Sexuality, Contested Space and Feminist Activism* (Durham: Duke University Press, 2007) and F. Enke, ‘Smuggling Sex Through the Gates: Race, Sexuality and the Politics of Space in Second Wave Feminism’, *American Quarterly*, 2003, 55, 639

67 Watkins, *On the Pill*, 54.

68 Interview with Ann H. (10 Jul 2018), 3.

69 M. D. Moore, ‘‘Waiting for the Doctor’: Managing Time and Emotion in the British National Health Service, 1948–1980’, *Twentieth Century British History*, 2022, 33, 212

70 Interview with Agnes (24 Nov 2020), 8.

71 Shena, 19.

72 Interview with Margaret (9 Aug 2019), 16.

73 Interview with Deborah (23 Nov 2020), 9.

74 Deborah, 9.

75 O’Neill, ‘Education Not Fornication’, 89.

76 Agnes, 13.

77 O’Neill, ‘Education Not Fornication’, 87.

78 Jessie, 4.

79 A. Mold, *Making the Patient-Consumer*, 19.

80 Cook, *The Long Sexual Revolution*, 74.

81 J. Campbell Murdoch, ‘Sex and the Single Girl’, *British Medical Journal*, 1972, 235–6.

82 Davis, ‘The Medical Profession and Abortion Law Reform’, 153–5.

83
*Ibid.*, 236.

84 Shena, 11–12.

85 Alison, 10.

86
*Ibid.*, 11.

87 Margaret, 8.

88 Brook Information Education Publications: ‘Social and Emotional Aspects of Pregnancy in Teenagers’, 1978 [WT, SA/FPA/C/E/16/5/9].

89 Kate, 12.

90 Margaret, 8.

91 Mold, *Making the Patient-Consumer*, 69–88.

92 Deborah, 13.

93 Shena, 14.

94 Carol, 1.

95 Interview with Louise (9 Oct 2019), 157.

96 Kirsty, 5–19.

97 Nash and Kilday, *Shame and Modernity*, 4.

98 Interview with Edith (3 March 2021), 13.

99 Maggie, 13.

100 Sheena, 12.

101 Interview with Liz A. (2 Aug 2018), 2.

102 Jessie, 4.

103 Watkins, *On the Pill*, 40.

104 G. Davis, ‘The Medical Community and Abortion Law Reform’; O’Neill, ‘Abortion Games’.

105 G. Davis, ‘The Medical Community and Abortion Law Reform’, 156.

106 Nash and Kilday, *Shame and Modernity in Britain*, 4.

107 Ross and Solinger, *Reproductive Justice*, 47.

108 Geiringer, *The Pope and the Pill*, 8.

109 See, for instance, Callum Brown, *Religion and the Demographic Revolution*.

110 Mold, *Making the Patient-Consumer.*

111 See, for example, J. Nelson, *More Than Medicine: A History of the Feminist Women’s Health Movement* (New York: New York University Press, 2015) which focuses on the USA, or L. Kelly, *Contraception and Modern Ireland: A Social History c. 1922–92* (Cambridge: Cambridge University Press, 2023) on Ireland.

## Notes

Kristin Hay (she/her) is a Lecturer in Economic and Social History at the University of Glasgow. Her interests lie in the history of sex, gender, medicine and oral history, with a particular emphasis on reproductive health and rights activism in the nineteenth and twentieth centuries.

